# High-Throughput Sequencing Reveals Circulating miRNAs as Potential Biomarkers for Measuring Puberty Onset in Chicken (*Gallus gallus*)

**DOI:** 10.1371/journal.pone.0154958

**Published:** 2016-05-05

**Authors:** Wei Han, Yunfen Zhu, Yijun Su, Guohui Li, Liang Qu, Huiyong Zhang, Kehua Wang, Jianmin Zou, Honglin Liu

**Affiliations:** 1 National Chickens Genetic Resources, Poultry institute, Chinese Academy of Agricultural Science, Yangzhou, PR China; 2 College of Animal Science & Technology, Nanjing Agricultural University, Nanjing, PR China; Huazhong University of Science and Technology, CHINA

## Abstract

There are still no highly sensitive and unique biomarkers for measurement of puberty onset. Circulating miRNAs have been shown to be promising biomarkers for diagnosis of various diseases. To identify circulating miRNAs that could be served as biomarkers for measuring chicken (*Gallus gallus*) puberty onset, the Solexa deep sequencing was performed to analyze the miRNA expression profiles in serum and plasma of hens from two different pubertal stages, before puberty onset (BO) and after puberty onset (AO). 197 conserved and 19 novel miRNAs (reads > 10) were identified as serum/plasma-expressed miRNAs in the chicken. The common miRNA amounts and their expression changes from BO to AO between serum and plasma were very similar, indicating the different treatments to generate serum and plasma had quite small influence on the miRNAs. 130 conserved serum-miRNAs were showed to be differentially expressed (reads > 10, *P* < 0.05) from BO to AO, with 68 up-regulated and 62 down-regulated. 4829 putative genes were predicted as the targets of the 40 most differentially expressed miRNAs (|log2(fold-change)|>1.0, *P* < 0.01). Functional analysis revealed several pathways that were associated with puberty onset. Further quantitative real-time PCR (RT-qPCR) test found that a seven-miRNA panel, including miR-29c, miR-375, miR-215, miR-217, miR-19b, miR-133a and *let*-7a, had great potentials to serve as novel biomarkers for measuring puberty onset in chicken. Due to highly conserved nature of miRNAs, the findings could provide cues for measurement of puberty onset in other animals as well as humans.

## Introduction

Precocious puberty is a pathologic status for humans, and could result in serious impact on health and development, including increase obesity, diabetes and cancer risks [1]. Puberty onset is a complicated process that comprises many genes and regulatory factors. Recently, much progress has been made in identifying components that regulate puberty onset [2]. Notably, a set of genes including KISS1/GPR54 [3], TAC3/TACR3 [4] and LIN28 [5] are found to play important roles in initiating puberty. However, association analysis of variants in genes with puberty onset are often not consistent among different groups [6,7]. Furthermore, the conclusion suggesting that timing of puberty for girls initiated earlier than in the past has long been a controversial topic [8].One of the main reasons is due to lack of unanimous standards to measure puberty. This, in turn, limits the discovery of new factors related to puberty onset.

To accurately estimate puberty onset is challenging [9]. In the last decade, there was no new measures of puberty emerged [8]. Physical alterations, thelarche, pubarche as well as age at menarch described by Tanner and vaginal opening [10] are the primary indicators used to measure female puberty onset in humans and mammals respectively. These are not accurate and efficient markers. Some blood reproductive hormones have also been widely applied to measure puberty, but due to the pulsatile secretion manner, their levels fluctuate easily and usually could not match to a specific pubertal stage [11,12]. So, to find new type biomarkers with higher sensitivity and specificity for puberty onset measurement is necessary.

microRNAs are small non-coding RNA molecules that suppress gene expression post-transcriptionally, and function important roles in diverse biological processes [13]. In addition to endogenous presence in cells, miRNAs can also be actively released into extracellular fluids through exosomes or microvesicles [14,15]. These circulating miRNAs can hold stably in body fluids, not only connect a new cell to cell communication mechanism but also their expression pattern changes are linked to body physiological and pathologic status. A number of circulating miRNAs have been identified as accurate biomarkers for disease diagnosis [16,17,18,19,20] and even for milk quality control [21,22]. This sheds light on measurement of puberty onset.

As an important model organism, chicken’s sexual development is very distinctive when compared to mammals and humans [23]. In our previous study, we had roughly determined the timing of transition from juvenility to puberty onset in Wenchang chicken, a Chinese indigenous chicken breed, through measuring inner gonad development and visible crown growth [24]. In order to find circulating miRNA biomarkers that could be served as biomarkers for measuring puberty onset in Wenchang chicken, in this study, we investigated the expression profile of miRNAs in the serum and plasma of chicken during puberty onset. The results demonstrate that chicken serum and plasma contains large amounts of miRNAs. A seven-miRNAs panel is identified as potential biomarkers for measuring chicken puberty onset. Considering the characteristics of miRNA functional conservation, the results will contribute to measurement of puberty onset in other animals as well as humans.

## Materials and Methods

### Ethics statement

The Wenchang chicken breed used in the present study is not endangered. The animals were allowed access to feed and water freely. Before sacrifice, the chickens were anaesthetized by giving them water mixed with diazepam and ethanol. When they became unconscious, the electric shocks were carried to minimize suffering. All animal experiments were approved by the Poultry institute, Chinese Academy of Agricultural Science, Yangzhou, China and Institutional Animal Care and Use Committees in College of Animal Science & Technology, Nanjing Agricultural University, Nanjing, China.

### Collection of serum and plasma samples

To generate serum, 4.0~5.0ml venous blood was collected into tubes free of anticoagulant. The whole blood was allowed to stand for 1 hour at 4°C before being centrifuged at 300×g for 5 minutes, and the supernatant by a final centrifugation at 800×g for 10 minutes.

To generate plasma, 4.0~5.0ml blood was collected into tubes with EDTA, directly centrifuged at 300×g for 5 minutes to spin down the blood cells, then followed by a second centrifugation at 800×g for 10 minutes to completely remove cellular components. The resultant serum and plasma were aliquoted into eppendorf tubes and stored at -80°C. All the centrifugation steps were performed at 4°C. Blood sample was processed and serum or plasma was frozen within 2 hours after blood was derived.

### Small RNA library preparation and sequencing

Collected serum and plasma from 6 hens at the age of 13 weeks were used to construct two small RNA libraries in this study. The 6 animals, including 3 BO (before puberty onset) ones and 3 AO (after puberty onset) ones, were from a mating family as described in detail previously [24]. Each library contained one full-sib and two half-sibs.

Total RNA was extracted from the serum and plasma using TruSeq Small RNA Sample Pre Kits (Illumine, San Diego, USA) according to the manufacturer’s instructions. Total RNA quality was checked with a Bioanalyzer 2100 (Agilent Technologies, USA). The RIN was > 8.0 and A260/A280 was > 2.1 for all samples. Total sera or plasma RNA of chickens from the same status were mixed with equal amounts to construct two pooled libraries. The overall flow of the sequencing procedure is as follows: small RNAs ranging from 18 to 35nt in length was purified from 15% polyacrylamide gels, then ligated to 5^,^ and 3^,^ adapters. Reverse transcription was performed, and followed by PCR amplification. The purified PCR products (~140bp) were used directly for cluster generation and sequencing analysis using the Illumina’s Solexa Sequencer according to the manufacturer’s instructions (LC-Bioscience, Hangzhou, China).

### Sequence data analysis

Sequence data analysis was done using AGGT101-miR tool. After deleting poor quality reads, adaptor pollution reads and reads less than 18nt, the clean reads were obtained.

The clean reads of small RNAs were aligned to the reference chicken (*G*.*gallus*) genome to identify known miRNAs. The sequences that matched perfectly to known miRNAs (miRBase V21.0) were determined as conserved miRNAs. Other small RNAs (rRNA, tRNA, snRNA and snoRNA) were annotated by blasting against the Rfram, Repbase and ncRNA databases.

The unannotated small RNA sequences were aligned to the reference chicken (*G*.*gallus*) genome to find potential precursor sequences for novel miRNAs. Novel miRNAs were predicted by RNA-fold tools following the criteria: (1) number of nucleotides in one bulge in stem (< = 12); (2) number of base pairs in the stem region of the predicted hairpin (> = 16); (3) cutoff of free energy (kcal/mol< = -15); (4) length of hairpin (up and down stems + terminal loop> = 50); (5) length of hairpin loop (< = 20); (6) number of nucleotides in one bulge in mature region (< = 8); (7) number of biased errors in one bulge in mature region (< = 4); (8) number of biased bulges in mature region (< = 2); (9) number of errors in mature region (< = 7); (10)number of base pairs in the mature region of the predicted hairpin (> = 12); (11)percent of mature in stem (> = 80). Furthermore, the raw reads > = 10 at least one time point.

To identify differentially expressed miRNAs, the number of conserved miRNAs was normalized to the total number of reads in each sample that matched the chicken (*G*.*gallus*) genome. P-values for differentially expressed miRNAs (Serum:BO vs AO; Plasma:BO vs AO) were calculated by Fisher^,^s exact-test and Chi square (2×2) test.

### Quantitative real-time PCR analysis

The candidate miRNAs for further quantitative real-time PCR (RT-qPCR) analysis were selected as the following criteria: (1) in the list of differentially expressed serum-miRNAs; (2) with more than middle expression levels (raw reads> = 40); (3) association with reproduction events, including gonad development, muscle development, glucose metabolism, fat metabolism, insulin metabolism, sex hormone synthesis and secretion.

Total RNAs of sampled sera and plasma were reverse-transcribed by PrimeScript® RT reagent Kit (TAKARA, DRR037A). The primers were designed by Primer 5.0 (ABI). 5ul RT reaction system included: denatured RNA and RT primer (2 uM) 3.0ul, 5×PrimeScript®Buffer 1.0ul, RNase Free dH2O 0.6ul, PrimeScript® RT Enzyme Mix I 0.4ul. The RT reactions were performed as follows: 42°C for 15 minutes, 85°C for 5 seconds and hold at 4°C. 20ul real-time PCR reaction system included: 2×SYBR Green Mix With ROX 10.0ul, ddH2O 8.2ul, Primer mix (10 uM) 0.8ul, RT product 1ul. The PCR reactions were performed as follows: 50°C for 2 minutes, 95°C for 2 minutes, then 40 cycles with 94°C for 15 seconds and 60°C for 30 seconds.

All experiments were performed on ABI 7900 HT sequence detection system. Each reaction was carried out with 3 replicates. snRNA U6 was used as the control for RT-qPCR. The relative expression level of each miRNA to U6 snRNA was normalized as ΔCq = Cq miRNA-Cq U6RNA [25]. Comparison of relative expression level in different stages was determined using the 2^-ΔΔCq^ method [26]. Statistical significance analysis of the expression change was performed by one-way ANOVA in SPSS 20.0.

### MiRNA target prediction and functional analysis

Target genes of differentially expressed miRNAs were predicted by TargetScan and miRanada. To acquire a higher prediction accuracy, only common target genes were considered. Gene Ontology (GO) annotation and Kyoto Encyclopedia of Genes and Genomes (KEGG) pathway analysis were retrieved using DAVID (http://david.abcc.ncifcrf.gov/).

**Fig 1:**
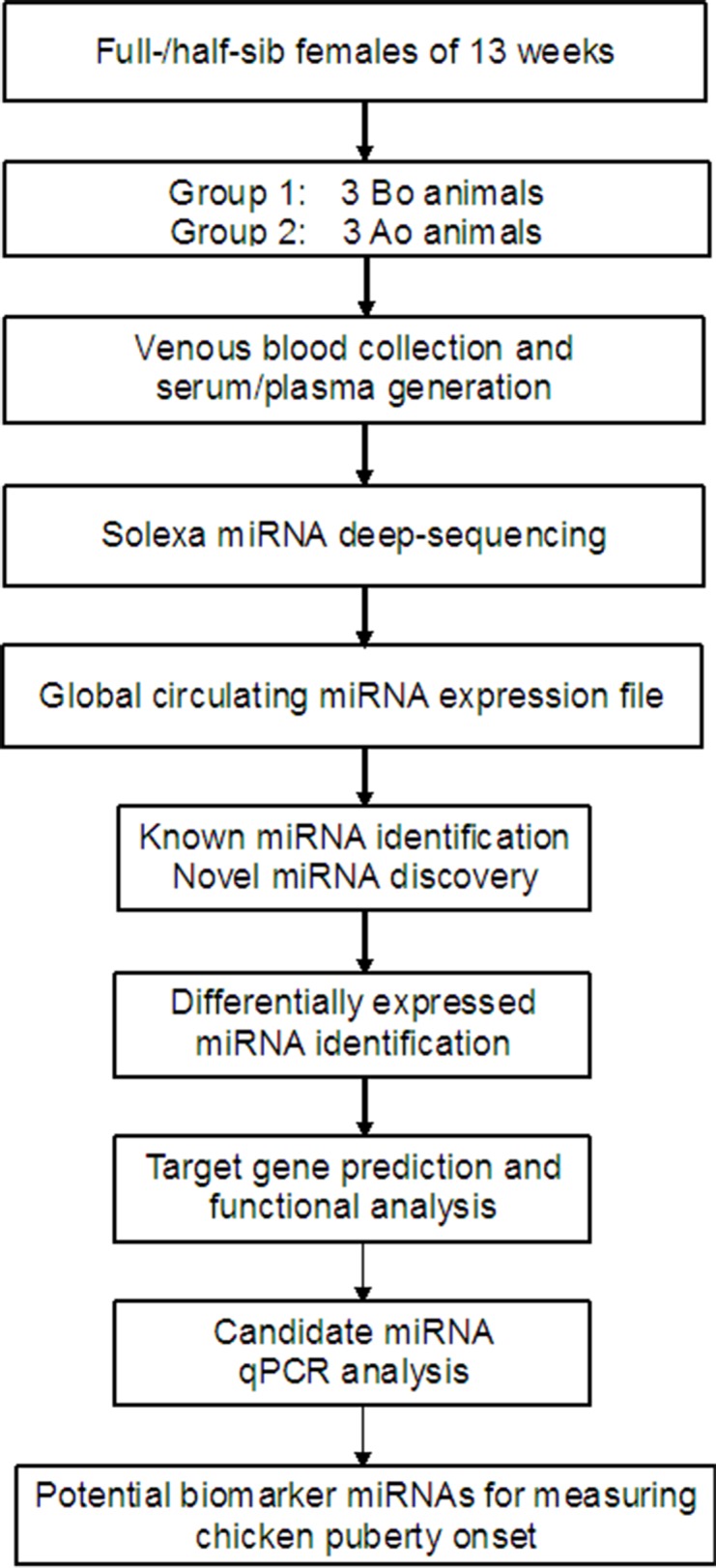
Global work chart of identification of potential circulating miRNAs for measuring chicken puberty onset. BO, before puberty onset; AO, after puberty onset.

The global work flow above was showed as Fig 1.

## Results

### Small RNA library construction and sequencing

**Fig 2:**
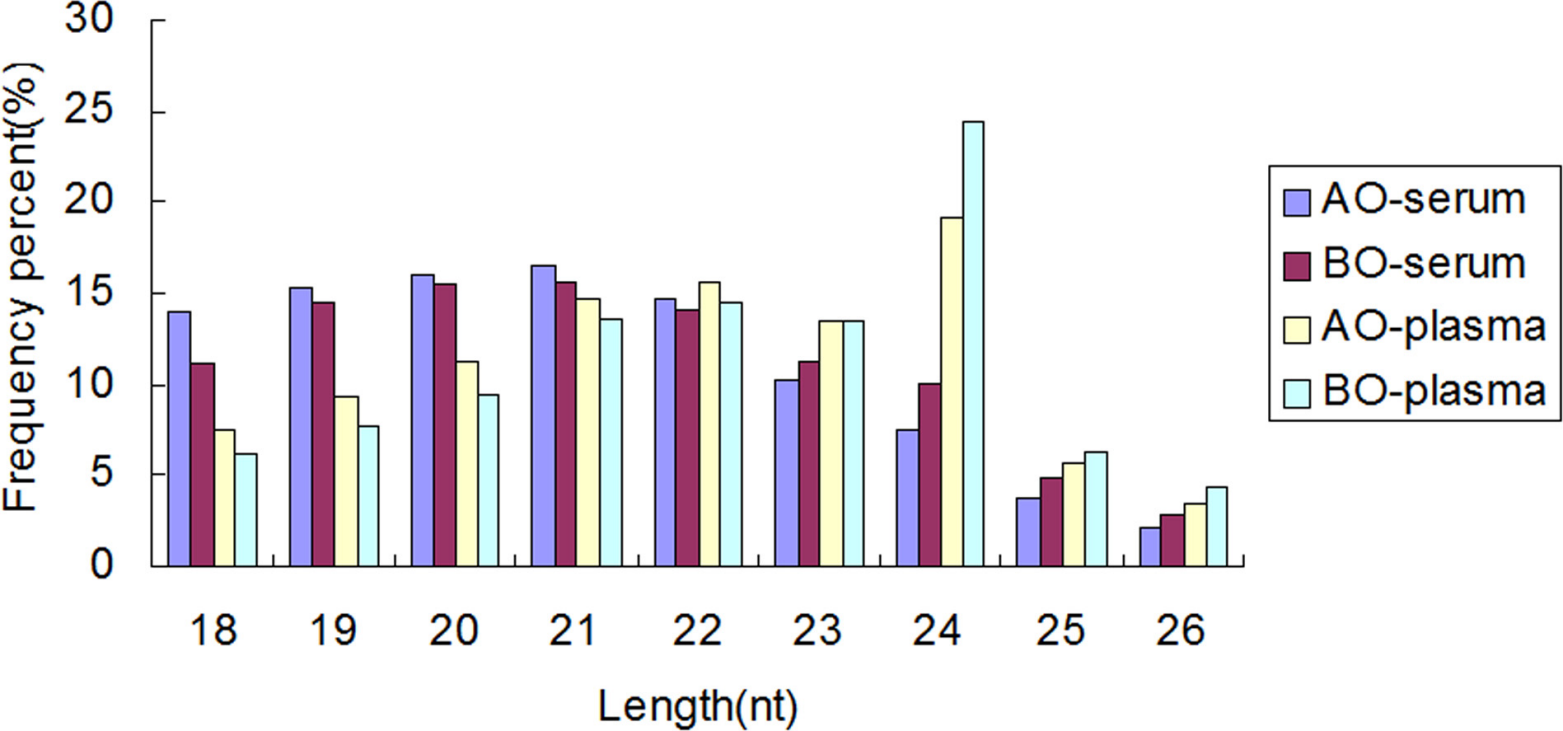
Frequency distribution of the sequence lengths in serum and plasma. The X axis shows sequence size. The Y axis shows the percents of sequence lengths. The majority of sequences for both libraries were 20nt ~ 24nt. BO, before puberty onset; AO, after puberty onset.

To investigate the miRNA expression profile in chicken serum and plasma, two small RNA libraries representing before puberty onset (BO) and after puberty onset (AO) status were constructed from full-/ half-sib animals. About 1.5~2.0 ml serum/plasma for each animal was used for RNA extraction, the RNA concentration was 30~40ng/ml. For each pool, RNAs were mixed at equal concentration (150ng of total RNA). High-throughput Solexa sequencing yielded 13,566,503 (BO) and 8,736,644 (AO) raw reads for serum small RNA libraries, and 9,522,247 (BO) and 10,082,482 (AO) raw reads for plasma small RNA libraries. After filtered low quality sequences, 7,552,415 (BO) and 4,268,347 (AO) clean reads for serum, 5,960,124 (BO) and 6,694,759(AO) clean reads for plasma were obtained respectively. The histograms of the reads length distribution showed majority were 20nt ~ 24nt (Fig 2). Of these, 205,107 (BO) and 218,200 (AO) unique small RNAs for serum, 214,226 (BO) and 221,870 (AO) unique small RNAs for plasma were identified.

### Identification of conserved miRNAs

To identify conserved miRNAs in chicken serum and plasma, the small RNAs were aligned to current miRBase (Release V21.0). Sequences with perfect matching to known chicken (*G*.*gallus*) miRNAs were considered as conserved miRNAs. In total, 398 conserved sequences were annotated as chicken miRNAs. To obtain higher reliable results, only the miRNAs with raw reads >10 at least one time point were considered. Then 192 were sorted as common miRNAs, with only 1 serum-specific miRNAs and 4 plasma-specific miRNAs. All 197 conserved miRNAs detected by sequencing were listed in S1 Table.

### Discovery of novel miRNAs

To predict novel miRNAs, the unannotated reads were analyzed using RNAfold tools. A total of 100 novel miRNAs expressed in serum or plasma met the predictive criteria. The abundance of these novel miRNAs was relatively lower, only 19 ones with raw reads >10 at least one time point (S2 Table). So, this might be the reason they were not found previously.

### Differential expression profiles of conserved miRNAs between BO and AO

To compare the differential expression of miRNAs in the serum/plasma of BO versus AO chickens, the number of miRNAs in each sample was normalized to the total number of reads. It was of note that the list of differential expression miRNAs in plasma was the same with that in serum. The expression of 4 plasma-specific miRNAs was not significant in AO chickens compared to BO counterparts. So only the differential expression miRNAs in serum were showed in S3 Table. In total, 130 miRNAs were considered to be differentially expressed (*P* < 0.05), with 68 up-regulated and 62 down-regulated. 40 miRNAs had more than two fold expression changes (|log2(fold-change)|> = 1.0) from BO to AO (Table 1).

**Table 1:** The differentially expressed serum-miRNAs with more than two fold-changes between BO and AO.

miRNAs	Fold-change	Log2(fold-change)	p-value
gga-miR-3536	16.48	4.04	< 0.001
gga-miR-499-3p	13.73	3.78	< 0.001
gga-miR-24-5p	12.09	3.60	< 0.001
gga-miR-27b-5p	6.04	2.60	0.005
gga-miR-2188-5p	5.83	2.54	< 0.001
gga-miR-193b-5p	5.60	2.49	< 0.001
gga-miR-3528	5.37	2.42	< 0.001
gga-miR-451	4.25	2.09	< 0.001
gga-miR-1736-3p	4.21	2.07	< 0.001
gga-miR-365-5p	3.85	1.94	< 0.001
gga-miR-144-3p	3.83	1.94	< 0.001
gga-miR-1552-3p	3.57	1.84	0.007
gga-miR-92-5p	3.30	1.72	< 0.001
gga-miR-365-3p	3.14	1.65	< 0.001
gga-miR-193b-3p	2.95	1.56	< 0.001
gga-miR-221-5p	2.75	1.46	0.011
gga-miR-142-3p	2.58	1.37	< 0.001
gga-miR-183	2.38	1.25	< 0.001
gga-miR-29b-3p	2.32	1.22	< 0.001
gga-miR-101-5p	2.30	1.20	< 0.001
gga-miR-1451-5p	2.16	1.11	< 0.001
gga-miR-142-5p	2.15	1.11	< 0.001
gga-miR-19a-3p	2.00	1.00	< 0.001
gga-miR-216b	0.50	-1.01	< 0.001
gga-miR-216a	0.49	-1.02	0.024
gga-miR-1664-3p	0.46	-1.13	< 0.001
gga-miR-375	0.45	-1.16	< 0.001
gga-miR-193a-3p	0.41	-1.30	0.003
gga-miR-193a-5p	0.38	-1.40	< 0.001
gga-miR-449c-5p	0.34	-1.54	< 0.001
gga-miR-31-5p	0.33	-1.60	0.002
gga-miR-217-5p	0.24	-2.04	< 0.001
gga-miR-20a-3p	0.24	-2.09	< 0.001
gga-miR-133a-3p	0.24	-2.05	< 0.001
gga-miR-155	0.22	-2.15	< 0.001
gga-miR-460b-5p	0.17	-2.53	< 0.001
gga-miR-29c-3p	0.15	-2.70	< 0.001
gga-miR-9-5p	0.15	-2.70	< 0.001
gga-miR-19b-3p	0.15	-2.80	< 0.001
gga-miR-460b-3p	0.09	-3.45	< 0.001

BO indicated before puberty onset, AO indicated after puberty onset.

### Target prediction and functional analysis of differential expression miRNAs

To further explore the roles of differentially expressed miRNAs, putative target genes of the most differentially expressed 40 miRNAs (|Log2 (fold-change)|> = 1.0) were predicted by integrating TargetScan and miRanda. In total, 4829 common target genes were found (data is not shown).

GO annotation (S4 Table) showed the putative target genes were significantly enriched (counts > 30, *P* < 0.05) in protein transport and protein catabolism biological processes. The KEGG analysis (S5 Table) suggested that MAPK signaling pathway, focal adhesion, regulation of actin cytoskeleton, endocytosis, ubiquitin mediated proteolysis and calcium signaling pathway were the most enriched pathways (counts > 50, *P* < 0.01).

### RT-qPCR validation of candidate miRNAs

**Fig 3:**
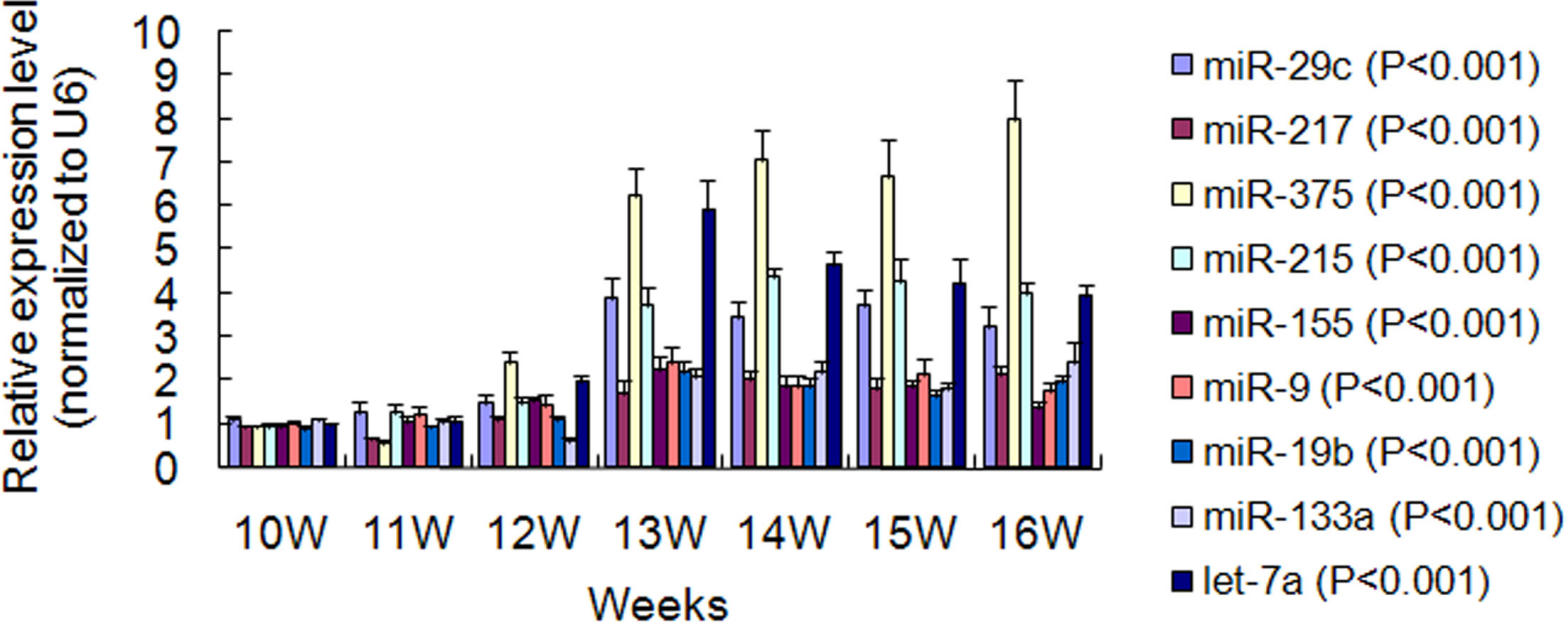
Relative expression changes of 9 candidate serum-miRNAs in different stages. Bars show standard deviations for replicates (n = 6).

To identify miRNAs that can be served as potential biomarkers for measuring puberty onset in chicken. RT-qPCR validation of 9 candidate miRNAs was performed in serum from 10 to 16 weeks. The primers were listed in S6 Table. The results demonstrated that expression of control U6 was very stable, with quantification cycle (Cq) difference between groups less than 0.6. The Cq of the 9 miRNAs also had smaller variation between samples in one group (S7 Table). The single peak in dissociation curve indicated higher specificity of PCR products. The melting temperature (Tm) was 80.0~90.0°C. As illustrated in Fig 3, 7 miRNAs including miR-29c, miR-217, miR-375, miR-215, miR-19b, miR-133a and *let*-7a had relatively low and stable expression levels (*P* < 0.05) in early period, and increased significantly (*P* < 0.01) from 12 to 13 weeks when the gonads entered into rapid development. More importantly, the increased higher expression levels for these 7 ones could keep or show further increment until age at the first egg. Although the expression levels of miR-155 and miR-9 also increased significantly (*P* < 0.01) from 12 to 13 weeks, they then dropped significantly (*P* < 0.05, *P* < 0.01) and recovered to lower levels as early period.

## Discussion

It has been suggested that circulating miRNAs are derived from multiple tissues. Specific miRNAs are enriched in exosomes in a cell-type-dependent manner [14]. miRNAs are found to express widely in gonad tissues and function roles in reproductive events [27,28,29,30]. Especially, the observations in model organisms and mammals have shown a potential link between miRNAs and puberty onset [31,32,33,34]. Our previous study also reveals miRNAs as novel partners involved in chicken puberty onset [24].This supports the possibility of developing circulating miRNA biomarkers to measure puberty onset.

The Solexa deep sequencing was performed to analyze the miRNA expression profiles in serum and plasma of hens from two different pubertal stages, before puberty onset (BO) and after puberty onset (AO). In total, 197 conserved miRNAs were identified in chicken serum and plasma. The co-expressed miRNA amounts (192/197) and their expression trends from BO to AO between serum and plasma were very similar, indicating that the different treatments to generate serum and plasma had nearly no influence on miRNAs. Many of our detected miRNAs had been found to express in various tissues, which further confirmed the wide origins of circulating miRNAs. Interestingly, some hypothalamic miRNAs involved in timing chicken puberty revealed by our previous study [24] were also abundantly expressed in serum and plasma. A recent report has confirmed small non-coding RNAs can transfer through mammalian placenta and directly regulate fetal gene expression [35]. Rom *et al* (2015) [36] found miR-98 and let-7g were protectors of the blood-brain barrier under neuroinflammatory conditions. Yet, whether these miRNAs are derived from the hypothalamus needs further investigation.

40 differentially expressed miRNAs were found during transition from BO to AO. To knowledge the global networks regulated by these differential miRNAs, target genes prediction and functional analysis were performed. The results suggest these differentially expressed miRNAs are involved in various biological pathways. Prominent among the pathways, MAPK signaling pathway has been suggested to play roles in female sexual maturation [37]. Wnt signaling pathway is believed to be a significant contributor to the regulation of ovarian follicle maturation and steroidogenesis [38]. mTOR signaling pathway is found to couple the nutritional status to regulate puberty onset in rats [39]. ErbB and GnRH signaling pathway is required for the timely initiation of puberty [40].

To identify circulating miRNAs can be served as biomarkers for measuring puberty onset in chicken is our main focus. The complex events involved in initiation of puberty require functional changes not only in neuroendocrine regulation genes and gonadal development genes, but also in energy metabolism-related genes. Therefore, there should be multiple miRNAs targeting those genes involved in puberty onset. Circulating miRNAs can derive from the gonad themselves or from metabolic tissues. According to previous reports and our sequencing results, 9 miRNAs, including miR-29c-3p, miR-375, miR-215-5p, miR-9-5p, miR-19b-3p, miR-133a-3p, let-7a, miR-217-5p and miR-155 were determined as candidates. Firstly, these miRNAs are differentially expressed from BO to AO with more than middle expression levels. Those ideal BO-specific or AO-specific miRNAs are not chosen just because of their lower expression, which will be a limitation in practical test. Of course, this criterion (raw reads> = 40) had disadvantage. It may not be suitable for other species. Secondly, they are associated with reproduction. Of the 9 members, miR-217 and miR-375 are regulators of chicken ovary maturity [29]. The developmental change of let-7a in hypothalamus is suggested to result in puberty onset in rats [33]. miR-375 and let-7a also play roles in regulation of insulin sensitivity and glucose metabolism [41,42,43]. miR-29c is linked to function in superior ovulation rate and kidding rate [44], and is a signature under high glucose conditions and a biomarker for anabolic steroids treatment [45]. miR-155 is found to be a molecular switch that regulates fat metabolism [46]. Circulating miR-19b is a marker of fatty liver [47]. Furthermore, miRNA-217, miRNA-155, miR-19b and miR-9 have target genes that are associated with puberty onset, such as FSHR, LEPR and circadian clock genes. The combination of the 9 serum miRNAs should be more reliable and unique than a single miRNA for puberty onset measurement.

To further validate the applicability of the nine miRNAs, RT-qPCR assays were performed in a set of individual serum samples and at multiple developmental stages. Seven of the nine miRNAs, except for miR-155 and miR-9, had a relatively consistent expression in earlier developmental phases, increased significantly when puberty initiated and hold stably or showed further increment until laying the first egg. Accordingly, a panel of seven miRNAs was identified as potential biomarkers to measure puberty onset in chicken. One of the weakness in our study is that the effectiveness of the seven-miRNA panel has not been confirmed in other chicken breeds. In addition, use of the seven-miRNAs panel can not differentiate the developmental progress after the initiation of puberty.

## Conclusions

The present study is the first to examine the miRNA expression profile in the serum and plasma of chickens during puberty onset. The results show that numerous miRNAs are present in chicken serum and plasma. A seven serum-miRNA panel is identified as potential biomarkers for measuring chicken puberty onset. Due to highly conserved nature of miRNAs, the findings from our study will provide cues for other animals as well as human precocious puberty research.

## Supporting Information

S1 Table The conserved miRNAs expressed in the chicken serum and plasma. (XLS)

S2 Table Novel miRNAs expressed in the chicken serum and plasma. (XLS)

S3 Table The differentially expressed serum-miRNAs between BO and AO. The miRNAs with |log2(fold-change)|> 1.0 were indicated in bold.
(XLS)

S4 Table GO analysis of the putative target genes. Go terms with counts of genes > 30 were indicated in bold.
(XLS)

S5 Table KEGG analysis of the putative target genes. KEGG pathway with counts of genes > 50 were indicated in bold.
(XLS)

S6 Table The primer sequences for validation of 9 candidate miRNAs. (XLS)

S7 Table Cq means of 9 candidate miRNAs in different period serum. (XLS)
